# Optimized Cocktail Phenotyping Study Protocol Using Physiological Based Pharmacokinetic Modeling and *In silico* Assessment of Metabolic Drug–Drug Interactions Involving Modafinil

**DOI:** 10.3389/fphar.2016.00517

**Published:** 2016-12-27

**Authors:** Angela Rowland, Arduino A. Mangoni, Ashley Hopkins, Michael J. Sorich, Andrew Rowland

**Affiliations:** ^1^Department of Clinical Pharmacology, School of Medicine, Flinders UniversityAdelaide, SA, Australia; ^2^Precision Medicine Group, Flinders Center for Innovation in Cancer, School of Medicine, Flinders University AdelaideSA, Australia

**Keywords:** physiological based pharmacokinetic modeling, cocktail phenotyping, metabolic drug–drug interactions, modafinil, study protocol

## Abstract

*In vivo* cocktail pathway phenotyping (ICPP) is routinely used to assess the metabolic drug–drug interaction (mDDI) potential of new drug candidates (NDC) during drug development. However, there are a number of potential limitations to this approach and the use of validated drug cocktails and study protocols is essential. Typically ICPP mDDI studies assess only the impact of interactions following multiple postulated perpetrator doses and hence the emphasis in terms of validation of these studies has been ensuring that there are no interactions between probe substrates. Studies assessing the comparative impact of single and multiple doses of the postulated perpetrator have the potential to provide richer information regarding both the clinical impact and mechanism of mDDIs. Using modafinil as a model compound, we sought to develop an optimized ICPP mDDI study protocol to evaluate the potential magnitude and clinical relevance of mDDIs using a physiologically based pharmacokinetic modeling approach.

## Introduction

*In vivo* cocktail pathway phenotyping (ICPP) is a relatively new approach that facilitates the simultaneous, but independent, assessment of the activity of multiple metabolic pathways. ICPP is ideally suited for the assessment of the magnitude of *in vivo* metabolic drug–drug interactions (mDDIs) involving cytochrome P450 (CYP) enzymes (Snyder et al., 2014). Indeed, ICPP is routinely used to assess the mDDI potential of new drug candidates (NDC) during drug development. However, there are a number of potential limitations to this approach, such as the capacity for interactions between probe drugs, increased risk of adverse effects caused by probe drugs, and increased analytical complexity (Tanaka et al., 2003). As such, the use of validated drug cocktails and study protocols is essential.

Frye et al. (1997) the “Pittsburgh cocktail” was reported as the first validated *in vivo* cocktail for the assessment of CYP enzyme activities. Today, validated drug cocktails include the “Cooperstown” (Streetman et al., 2000a,b), “Karolinska” (Christensen et al., 2003), and “Inje” (Ryu et al., 2007) cocktails. Each cocktail has advantages and disadvantages, and in some cases they have been modified to expand their capacity (Chainuvati et al., 2003; Snyder et al., 2014) or address limitations. The four probe drugs in the Cooperstown cocktail; caffeine (CYP1A2), omeprazole (CYP2C19), dextromethorphan (CYP2D6), and intravenous midazolam (CYP3A), are generally considered the core ‘probes’ of the CYP cocktail approach. These drugs do not interact with each other and are not associated with significant adverse effects. However, the intravenous administration of midazolam (probe for CYP3A) precludes the assessment of gastrointestinal CYP3A enzyme activity. Furthermore, this cocktail does not contain a probe drug for CYP2C9, which accounts for approximately 18% of the CYP protein in human liver (Miners and Birkett, 1998). These limitations are partially ameliorated by using warfarin as a probe for CYP2C9 in the “Cooperstown 5+1” cocktail (Chainuvati et al., 2003). The Karolinska 5-drug cocktail uses caffeine (CYP1A2), losartan (CYP2C9), omeprazole (CYP2C19), debrisoquine (CYP2D6), and quinine (CYP3A) as probe drugs. However, the simultaneous administration of the cocktail drugs causes a significant increase of metabolic ratio of debrisoquine. Therefore, this approach requires the separate oral intake of debrisoquine, with additional logistical complexity. The “Inje” cocktail comprises caffeine (CYP1A2), losartan (CYP2C9), omeprazole (CYP2C19), dextromethorphan (CYP2D6) and oral midazolam (CYP3A). These probe drugs do not interact with each other and are not associated with significant adverse effects. Furthermore, the use of these drugs as probes facilitates the assessment of the effects of mDDIs on all major drug metabolizing CYP enzymes including both hepatic and gastrointestinal CYP3A.

Historically ICPP based mDDI studies have only assessed the impact of interactions following multiple (3 to 5) doses of the postulated ‘perpetrator,’ where an interaction perpetrator is a compound that has the potential to alter the metabolic clearance of other drugs. As such, the emphasis in terms of validation of these studies has been ensuring that there are no interactions between probe substrates. Increasingly, however, it is recognized that the mechanism of mDDIs both in terms of inhibition (i.e., mechanism based versus reversible) and activation (i.e., induction of enzyme expression versus enhanced substrate binding) of CYP activity can have important clinical implications. While multiple dose mDDI studies are useful in describing the effect of steady state dosing of the postulated perpetrator on CYP activity, they provide limited insights regarding the mechanism of mDDIs. Studies assessing the comparative impact of single and multiple doses of the postulated perpetrator, providing information both on the clinical impact and mechanism of mDDIs, have not been routinely performed and appropriate validated dosing protocols to facilitate such studies remain to be defined.

Modafinil, a drug able to both induce and inhibit CYP activities *in vitro*, particularly CYP3A (Robertson et al., 2000), is an ideal candidate to assess the comparative impact of single dose versus steady state dosing on CYP activity using an ICPP mDDI study design. We sought to develop an optimized ICPP mDDI study protocol to evaluate the potential magnitude and clinical relevance of mDDIs perpetrated by modafinil using a physiologically based pharmacokinetic (PBPK) modeling approach.

## Methods

### Structural Model

Simulations were performed using the Simcyp^®^ Simulator (version 15.1; Jamei et al., 2009) with a ‘minimal PBPK model’ comprising a liver compartment and a merged compartment representing all other organs (Howgate et al., 2006; Polasek et al., 2009; Wattanachai et al., 2011). The differential equations used by the simulator describing enzyme kinetics and the impact of co-variates have been described previously (Rowland Yeo et al., 2010).

### Population Model

Unless specified otherwise, simulations were performed using the Simcyp Healthy Volunteer population profile with virtual study cohorts comprising an equal distribution of healthy males and females aged 21–40 years old. Simulation data are presented as the geometric mean and 95% confidence interval (CI) for 120 estimations (10 studies of 12 individuals).

### Development of Modafinil Model

#### Compound (Substrate and Inhibitor) Profile

**FIGURE 1:**
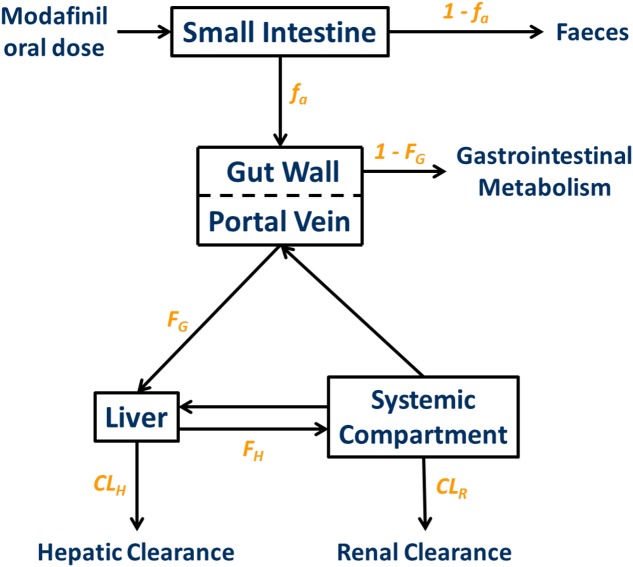
**Minimal physiologically based pharmacokinetic (PBPK) model used to simulate and predict the pharmacokinetics of modafinil.** *f*_a_, fraction absorbed; *k*_a_, absorption rate constant; *F*_G_, fraction escaping gastrointestinal metabolism; *F*_H_, fraction escaping hepatic metabolism; CL_H_, hepatic clearance; CL_R_, renal clearance.

The physicochemical, blood binding, absorption, distribution, elimination, and interaction data used to construct the modafinil compound (substrate and inhibitor) profiles are summarized in **Table 1**. A schematic depicting the minimal PBPK model used to simulate and predict the pharmacokinetics of modafinil is shown in **Figure 1**. All parameters were based on published literature values or were model predicted based on the physicochemical characteristics of the drug. Modafinil hepatic microsomal intrinsic clearance (CL_int_) was back-calculated from clinically observed oral clearances (CL_PO_; Wong et al., 1998a) using the retrograde model function in Simcyp (Yamazaki et al., 2015). The interaction (‘inhibitor’) profile was created based on published *in vitro* microsomal inhibition and hepatocyte induction data for CYP 1A2, 2C9, 2C19, 2D6 and 3A4 (Robertson et al., 2000). The presence of endogenous fatty acids in microsomal preparations is known to effect the *in vitro* determination of kinetic parameters for multiple CYP enzymes including CYP 1A2, 2C9 and 2C19 (Rowland et al., 2008; Wattanachai et al., 2012, 2015). As such, published *K*_i_ values for CYP 1A2, 2C9, and 2C19 were scaled based on the known effects of endogenous fatty acids on these enzymes.

**Table 1:** Substrate and inhibitor parameter values used for modafinil substrate profile.

Physiochemical properties	
Molecular weight	273.4
log P_o:w_	1.53
p*K*_a_ (monoprotic base)	8.84
B/P (SimCYP predicted)	0.887
*f*_up_	0.4 (Wong et al., 1998a)

***In vivo* pharmacokinetic properties**	

*f*_a_ (Simcyp predicted)	0.99
*k*_a_ (1/h; Simcyp predicted)	3.93
*Q*_gut_ (L/h; Simcyp predicted)	15.56
*V*_ss_ (L/kg; Simcyp predicted)	0.72
CL_po_ (L/h)	3.23 (Wong et al., 1998a)

***In vitro* inhibition parameters (*K*_i_;μM; Robertson et al., 2000)**	

CYP1A2	750
CYP2C9	750
CYP2C19	7.8
CYP2D6	1,500
CYP3A4	632

***In vitro* induction parameters (IndC_50_; μM/Ind_Max_; Robertson et al., 2000)**	

CYP1A2	75/1.9
CYP2C9	N/A
CYP2C19	N/A
CYP2D6	N/A
CYP3A4	10/2.5

*P_o:w_, neutral species octanol: buffer partition coefficient; B/P, blood-to-plasma partition ratio; f_up_, fraction unbound in plasma; f_a_, fraction available from dosage form; k_a_, first-order absorption rate constant; V_ss_, volume of distribution at steady state; CL_po_, oral clearance; K_i_, inhibition constant; IndC_50_, inducer concentration that causes half maximal induction; Ind_Max_, maximal fold induction.*

#### Validation of Substrate Profile

The modafinil substrate profile (simulated exposure) was validated by comparison of the pharmacokinetics parameters maximal plasma concentration (*C*_Max_), time to maximal plasma concentration (*t*_Max_), area under the plasma concentration time curve (AUC) and elimination half-life (*t*_1/2_) from simulations with data from age and gender matched participants from three clinical studies (*n* = 6–12) that were not used in the development of the modafinil compound profile (Wong et al., 1998a, 1999b; Robertson and Hellriegel, 2003).

## Stepwise Procedure

### Compound Profiles

Simulations performed to assess CYP 1A2, 2D6, 3A4 and 2C19 activities used validated Simcyp substrate profiles for caffeine, dextromethorphan, midazolam and omeprazole, respectively (Jamei et al., 2009). Simulations performed to assess CYP2C9 activity used a losartan substrate profile based on published parameters (Brantley et al., 2014). Probe doses were 100 mg orally (PO) of caffeine, 25 mg PO of losartan, 20 mg PO of enteric coated omeprazole, 30 mg PO of dextromethorphan and 1 mg PO of midazolam.

### Validation of Study Dosing Protocol

**FIGURE 2:**
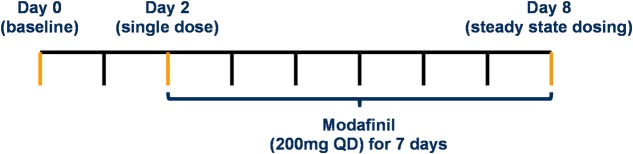
**Day 0, 2 and 8 protocol for the assessment of modafinil as a perpetration of mDDIs**.

The study dosing protocol was validated using PBPK modeling in terms of the minimal washout period between probe doses to facilitate assessment of CYP activity under three conditions (baseline, single modafinil dose and steady state modafinil dosing) and the minimal number of modafinil doses to achieve steady state. Following a series of preliminary simulations, two dosing protocols were assessed in full; a “Day 0, 1, 7 protocol,” where probes were dosed at 0, 24, and 168 h, with assessment of exposure at 200 uniformly distributed sampling time points over 192 h, and a “Day 0, 2, 8 protocol,” where probes were dosed at 0, 48, and 192 h, with assessment of exposure at 200 uniformly distributed sampling time points over 216 h (**Figure 2**). Residual concentration at 0.5 h prior to the second dose, AUC and *C*_Max_ for each probe in the absence of modafinil dosing were compared between the protocols. Trough modafinil concentrations (0.5 h prior to the subsequent dose) were evaluated over 7 days to determine the minimal dosing duration required to achieve repeat trough concentrations within 5%.

### Simulated Modafinil ICPP mDDI Study

The simulated impact of a single dose of modafinil (200 mg; PO) administered 1 h prior to the second probe dose and steady state dosing of modafinil [200 mg PO once daily (QD) for 7 days] on caffeine, losartan, omeprazole, dextromethorphan and midazolam exposure was assessed using the Day 0, 2, 8 protocol. The capacity for modafinil to perpetrate mDDIs was predicted on the basis of the simulated probe AUC ratios, with and without modafinil (single dose or steady state). The geometric mean of the AUC ratio for each probe was estimated using a mixed effects model of logarithmically transformed data. Time period was included as a fixed effect and participant as a random effect. Back transformation was utilized to provide a point estimate and 95% CI for the AUC ratio. Based on an equivalence approach a lack of effect of modafinil on CYP activity was demonstrated if the 95% CI for the estimated AUC ratio for the probe was contained within the range 0.85 to 1.2.

## Anticipated (Simulation) Results

### Optimisation and Validation of ICPP mDDI Model

#### Modafinil Substrate Profile

**FIGURE 3:**
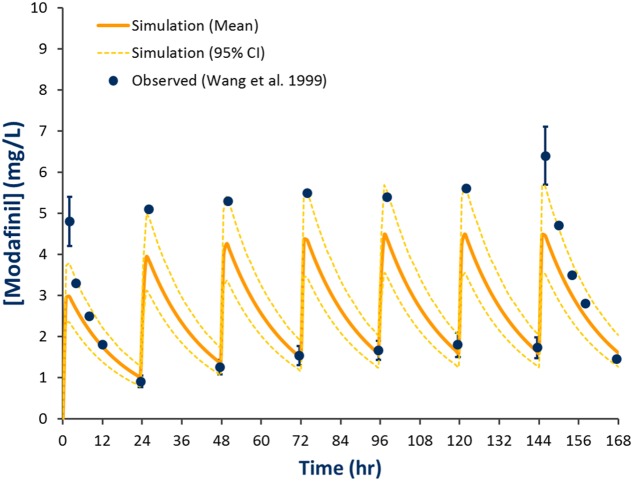
**Simulated and observed plasma concentration-time profiles defining modafinil exposure for 200 mg QD PO dosing for 7 days.** Solid line represents the mean model predicted exposure profile, broken line represents the 95% CI for the predicted exposure profile and dots represent observed data (±SD where reported; Wong et al., 1999b).

Comparison of simulated and observed pharmacokinetic parameters reported in **Table 2** demonstrate that the modafinil substrate profile accurately estimates exposure to this drug following a single dose (Wong et al., 1999a; Robertson and Hellriegel, 2003) and steady state dosing (Wong et al., 1999b). Simulated and observed (Wong et al., 1999b) plasma concentration-time profiles for modafinil (200 mg PO QD) over 7 days are shown in **Figure 3**. Estimated mean modafinil trough concentrations were 1.00, 1.30, 1.45, 1.47, 1.52, and 1.53 mg/L on days 2, 3, 4, 5, 6, and 7, respectively. Repeatable trough concentrations within 5% of the highest trough concentration were obtained from day 5 onwards. Simulations confirm that steady state exposure to modafinil is achieved within the 7 days perpetrator dosing period recommended by the Food and Drug Administration (FDA) for the assessment of induction and mechanism based inhibition mDDIs (FDA, 2012).

**Table 2:** Comparison of modafinil pharmacokinetics in age and gender matched simulations (mean and 95 % CI) and *in vivo* clinical studies.

Population (age range)	Dose	Study	*C*_Max_ (mg/L)	*t*_Max_ (h)	AUC (mg/L h)	*t*_1/2_ (h)
Males (22–37 y/o; Wong et al., 1999a)	200 mg	Observed	4.2	1.5	57	12
		Simulation	3.0 (2.8–3.1)	1.4 (1.3–1.4)	59 (55–62)	16 (14–17)
Females (19–40 y/o; Wong et al., 1999a)	200 mg	Observed	5.2	1.5	61	10
		Simulation	3.3 (1.3–1.4)	1.4 (1.3–1.4)	60 (57–64)	14 (13–15)
Males (20–39 y/o; Wong et al., 1999b)	200 mg QD for7 days	Observed	6.4	2.5	79	17
		Simulation	4.4 (4.2–4.7)	1.3 (1.2–1.4)	77 (72–83)	17 (16 –19)

*AUC, area under the plasma concentration-time curve; C_Max_, maximal plasma concentration; t_Max_, time taken to achieve maximal plasma concentration; t_1/2_, elimination half-life.*

#### Study Dosing Protocol

**FIGURE 4:**
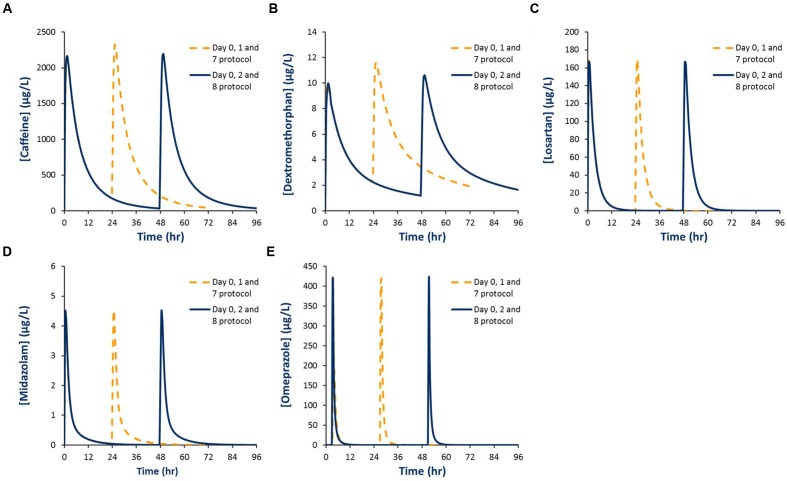
**Simulated plasma concentration-time profiles for Inje cocktail probes using two different probe dosing schedules. (A)** Caffeine; **(B)** Dextromethorphan; **(C)** Losartan; **(D)** Midazolam; **(E)** Omeprazole.

Baseline and second dose mean plasma concentration-time profiles for each probe obtained using the Day 0, 1, 7 and Day 0, 2, 8 protocols are shown in **Figure 4**. Corresponding residual concentration, AUC and *C*_Max_ values are described in **Table 3**. Simulations demonstrated significant residual caffeine and dextromethorphan concentrations prior to the second dose using the Day 0, 1, 7 protocols of 186 and 2.3 μg/L, respectively. Consistent with this observation, comparison of exposure (AUC and *C*_Max_) for probes dosed on Day 0 and Day 1 in the absence of modafinil, demonstrate that the second dose (Day 1) mean AUC and *C*_Max_ for caffeine were increased by 9.9 and 9.8 %, respectively, while the second dose (Day 1) mean AUC and *C*_Max_ for dextromethorphan were increased by 18.1 and 17%, respectively. Simulations performed using the Day 0, 2, 8 protocol demonstrated that estimated second dose (Day 2) mean AUC and *C*_Max_ values for all probes were within 5% of the corresponding first dose (Day 0) value. Mean AUC and *C*_Max_ values for probes administered 7 days after the second dose (i.e., Day 7 or 8) were also invariably within 5% of the first dose (Day 0) value with residual baseline probe concentrations of 0 μg/L (not shown).

**Table 3:** Comparison of mean (and 95% CI) pharmacokinetic parameters defining probe exposure between Day 0, 1 and 7 and Day 0, 2 and 8 dosing protocols.

Probe	Protocol	[Probe]_Residual_ (μg/L)		AUC (μg/L h)			*C*_Max_ (μg/L)		
		23.5 h	47.5 h	First dose	24–72 h	48–96 h	First dose	Day 2	Day 3
Caffeine	Day 0, 1, 7	186 (143–229)		16,970	18,653^#^(16,518–21,083)		2,128 (1,986–2,282)	2,337^#^(2,192–2,491)	
	Day 0, 2, 8		34 (26–45)	(14,868–18,961)		16,773 (14,858–18,936)			2,145
Dextromethorphan	Day 0, 1, 7	2.3 (1.8–2.9)		72 (59–88)	85^#^ (72–101)		9.9 (8.7–10.6)	11.6^#^ (10.7–12.9)	(2,003–2,299)
	Day 0, 2, 8		1.2 (0.8–1.6)			74 (61–89)			10.4 (9.3–12.4)
Losartan	Day 0, 1, 7	0.4 (0.0–0.9)		575 (529–624)	576 (530–625)		165 (152–178)	165 (152–178)	
	Day 0, 2, 8		0.0 (0.0–0.2)			575 (529–624)			167 (154–180)
Midazolam	Day 0, 1, 7	0.1 (0.0–0.2)		11.4 (10.1–13.0)	11.6 (10.2–13.3)		4.2 (3.8–4.7)	4.5 (4.1–4.9)	
	Day 0, 2, 8		0.0 (0.0–0.0)			11.4 (10.1–13.0)			4.5 (4.1–4.9)
Omeprazole	Day 0, 1, 7	0.0 (0.0–0.0)		391 (341–449)	391 (341–449)		420 (376–465)	420 (376–465)	
	Day 0, 2, 8		0.0 (0.0–0.0)			391 (341–449)			420 (376–465)

*^#^ Mean parameter estimate 10% greater than corresponding first dose value.*

### Assessment of Modafinil as an mDDI Perpetrator

**FIGURE 5:**
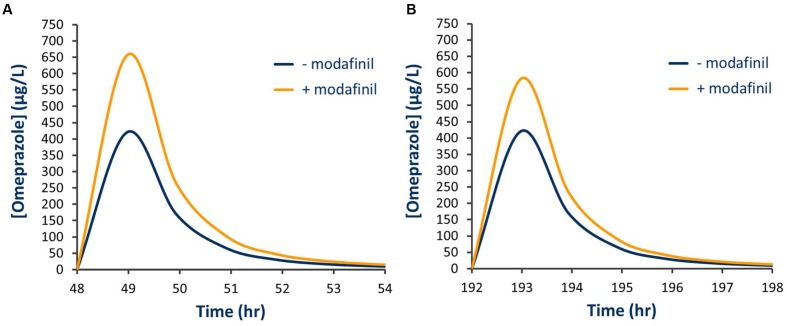
**Simulated plasma concentration time curve for omeprazole in the presence and absence of modafinil. (A)** Single modafinil dose; **(B)** Steady state modafinil dosing.

**FIGURE 6:**
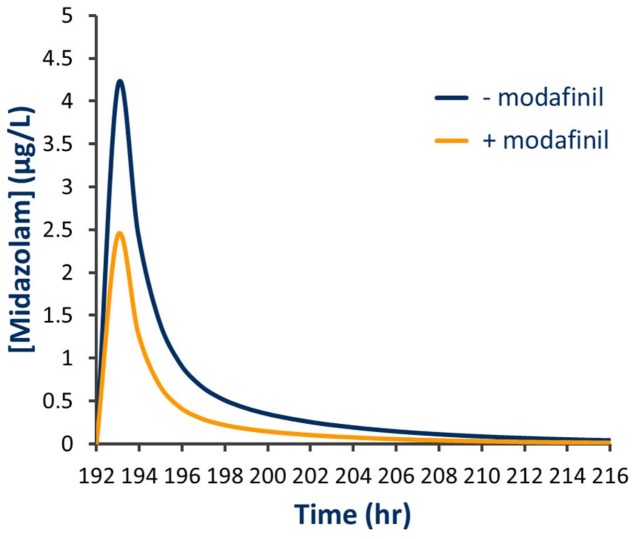
**Simulated plasma concentration time curve for midazolam in the presence and absence of steady state modafinil dosing**.

Simulated mean probe AUC values and ratios with 95% CIs in the presence and absence of a single dose of modafinil and steady state dosing of modafinil are shown in **Tables 4** and **5**, respectively. Following a single oral dose of modafinil, the mean AUC omeprazole increased by 56% (391 to 610 μg/L h). However, the magnitude of this predicted mDDI was partially attenuated following dosing of modafinil to steady state; 38% increase in omeprazole AUC from 391 to 455 μg/L h (**Figure 5**). These data indicate that modafinil may perpetrate clinically relevant inhibitory mDDIs when co-administered with drugs primarily metabolized by CYP2C19. The partially attenuated interaction simulated here following dosing of modafinil to steady state is likely due to an increase in CYP3A4 catalyzed omeprazole metabolism (see Discussion). Dosing of modafinil to steady state (7 days) resulted in a 50% decrease in midazolam mean AUC (11.2 to 5.6 μg/L h; **Figure 6**). These data indicate that modafinil causes clinically relevant induction of CYP3A4 with steady state dosing. No change in midazolam AUC was observed following a single dose of modafinil. Similarly, no significant effect of modafinil on CYP1A2 (caffeine), CYP2C9 (losartan) or CYP2D6 (dextromethorphan) was observed following either a single dose or dosing to steady state (probe drug Δ AUC < 5 %).

**Table 4:** Area under the plasma-concentration time curve (mean and 95 % CI) for probes in the absence and presence of a single dose of modafinil (200 mg PO).

Enzyme	Substrate (dose)	AUC (μg/L h)		
		Without modafinil	With modafinil	Ratio
CYP1A2	Caffeine (100 mg)	16,787 (14,867–18,956)	16,713 (14,806–18,865)	1.00 (0.99–1.00)
CYP2C9	Losartan (25 mg)	575 (529–624)	571 (526–620)	0.99 (0.99–1.00)
CYP2C19	Omeprazole (20 mg)	391 (341–449)	610 (537–691)	1.56 (1.52–1.59)
CYP2D6	Dextromethorphan (30 mg)	71.6 (58.8–87.2)	71.5 (58.9–86.8)	1.00 (0.99–1.00)
CYP3A4	Midazolam (1 mg)	11.4 (10.5–13.0)	10.9 (9.6–12.4)	0.96 (0.95–0.96)

**Table 5:** Area under the plasma-concentration time curve (mean and 95% CI) for probes in the absence and presence of modafinil (200 mg PO QD) dosed to steady state (7 days).

Enzyme	Substrate (dose)	AUC (μg/L h)		
		Without modafinil	With modafinil	Ratio
CYP1A2	Caffeine (100 mg)	16,556 (14710–18633)	15,941 (14166–17939)	0.96 (0.96–0.97)
CYP2C9	Losartan (25 mg)	573 (528–623)	526 (485–571)	0.92 (0.91–0.93)
CYP2C19	Omeprazole (20 mg)	391 (341–449)	455 (413–501)	1.38 (1.32–1.44)
CYP2D6	Dextromethorphan (30 mg)	70.2 (58.2–84.7)	68.5 (57.2–82.1)	0.98 (0.97–0.98)
CYP3A4	Midazolam (5 mg)	11.2 (9.9–12.8)	5.6 (4.9–6.4)	0.50 (0.47–0.53)

## Discussion

This study reports a validated dosing protocol for the administration of Inje cocktail probes that facilitates the assessment of the magnitude of potential mDDIs following a single perpetrator dose and steady state perpetrator dosing. Consistent with the estimated half-life of modafinil in healthy volunteers (∼15 h; Robertson and Hellriegel, 2003), simulated steady-state concentrations were obtained with 5 days of dosing. Repeatable simulated mean trough concentrations (defined as within 5%) were observed from Day 5 onwards. The minimal duration of perpetrator dosing considered sufficient to facilitate assessment of potential induction and mechanism-based inactivation of CYP enzymes e.g., time-dependent changes in CYP activity is 7 days (FDA, 2012). Simulations demonstrate that this dosing duration was also sufficient to ensure steady state modafinil concentrations.

In order to assess changes in CYP activity caused by the administration of the postulated mDDI perpetrator (single or steady state dosing), ICPP based mDDI studies require administration of probes prior to perpetrator administration to establish baseline CYP activities (Day 0). Simulations demonstrate that when assessing the impact of a single dose of the postulated mDDI perpetrator, repeat probe dosing on Day 1 (i.e., the Day 0, 1 and 7 protocol) is suitable for evaluation of CYP 2C9 (losartan), 2C19 (omeprazole) and 3A4 (midazolam) activities since no residual probe is present and AUC and *C*_Max_ values are equivalent to the first dose. However, this protocol is not suitable for assessment of CYP 1A2 (caffeine) or 2D6 (dextromethorphan) activities due to the presence of significant residual probe in the systemic circulation at the time of the second dose (24 h), leading to an overestimation of AUC and *C*_Max_ for these probes (**Table 3**). Use of this protocol will bias activity assessment toward inhibitory mDDIs involving CYP 1A2 and 2D6 increasing the risk of either detecting an inhibitory interaction (false positive) or failing to detect induction (false negative).

Simulations demonstrate that when assessing the single dose effect of the postulated mDDI perpetrator, administration of probes 48 h after the initial dose (Day 0, 2 and 8 protocol) is suitable for evaluation of all CYP activities since essentially no residual probe is present and AUC and *C*_Max_ values are comparable between the first and second dose (**Table 3**). On this basis, the Day 0, 2 and 8 protocol was used for the assessment of modafinil as a perpetrator of mDDIs involving CYP and is recommended for ICPP mDDI studies performed to assess single dose and steady state dosing effects of the a postulated mDDI perpetrator.

The capacity of modafinil to inhibit and induce CYP activity has been demonstrated *in vitro*. Classical (i.e., [I]/*K*_i_) extrapolation of *in vitro* data suggests that modafinil may induce expression of CYP 1A2 and 3A4 and inhibit CYP 2C19 and 3A4 activities in a reversible, competitive manner (Robertson et al., 2000). On the basis of these classically extrapolated *in vitro* data, modafinil is classified as a ‘moderate’ inducer of CYP 3A4 by the US FDA. Increasingly, however, it is accepted that there are several limitations (such as the appropriate selection of [I] (Rowland et al., 2006)) associated with the use of classical extrapolation of *in vitro* data when assessing mDDIs that preclude sensible assessment of the clinical relevance of novel interactions. Indeed, the substantial benefits of mechanistic (PBPK) extrapolation of mDDI assessments have been extensively reported and adopted (Sheiner and Steimer, 2000; Danhof et al., 2008; Rowland Yeo et al., 2010; Rowland et al., 2011) and this approach is recommended for the preclinical assessment of mDDIs for regulatory review (Zhao et al., 2011; FDA, 2012).

On the basis of the *in vitro* interaction profile (warfarin; CYP2C9; Robertson et al., 2002a), or potential for concomitant use with other vigilance (dexamphetamine, methylphenidate) or sedative (triazolam) agents (Wong et al., 1998b; Hellriegel et al., 2001, 2002; Robertson et al., 2002b), a limited number of *in vivo* studies assessing the mDDI potential of modafinil have been reported. Notably, these study ‘victim’ drugs, including warfarin, which was administered as a racemic mix or *r-* and *s*-enantiomers (metabolised by CYP 3A4 and 2C9, respectively) have been substrates for multiple metabolic pathways, and as such these studies do not readily facilitate the direct assessment of the effects of modafinil on individual CYP enzymes. Only one *in vivo* interaction study has reported the capacity of modafinil to induce drug metabolizing CYP (Moachon et al., 1996). This study used antipyrine, a pan-CYP substrate, with 7 days of modafinil dosing at 100 to 500 mg BD. The results of the study suggest that modafinil may be a weak general inducer of CYP at doses ≥400 mg/day (double the recommended daily dose), but no definitive conclusions were possible. The study design did not facilitate assessment of effects on individual CYP enzymes or account for concurrent inhibition of these enzymes.

**FIGURE 7:**
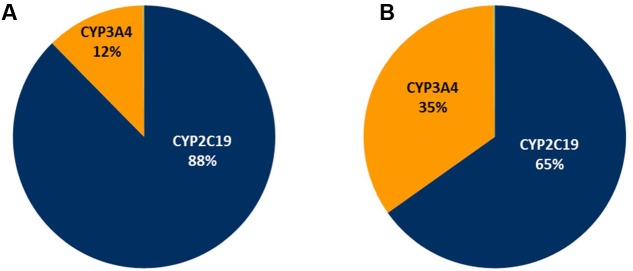
**Simulated omeprazole fraction metabolism (*f*_m_) pie charts. (A)** The absence of modafinil dosing; **(B)** Following dosing of modafinil to steady state.

In the current study, a clinically relevant increase in omeprazole exposure was predicted following both a single modafinil dose and dosing of modafinil to steady state. Notably, the apparent magnitude of the inhibitory interaction was attenuated with dosing of modafinil to steady state; omeprazole AUC ratios following a single modafinil dose and steady state modafinil dosing were 1.56 (1.52–1.59) and 1.38 (1.32–1.44), respectively. While omeprazole is primarily (88%) cleared by CYP2C19 catalyzed demethylation and hydroxylation, the remainder of omeprazole clearance occurs via CYP3A4 catalyzed sulfone formation. Following repeated dosing of modafinil, which induces CYP3A4 while inhibiting CYP2C19, the inhibitory effect on CYP2C19 is partially offset by induction of CYP3A4. The resulting change in fraction metabolized (*f*_m_) for omeprazole is shown in **Figure 7**. These data highlight both the advantages of mechanistic extrapolation of mDDI data when assessing complex interactions involving victim drugs that are metabolized by multiple enzymes (Rowland Yeo et al., 2010), and the need to consider even minor metabolic pathways when considering the selection of probe substrates as induction of a minor metabolic pathway can result in a significant contribution to clearance when induced. Where multiple enzyme involvement results in the formation of unique metabolites for each enzyme, this potential confounder may be overcome by the consideration of an enzyme specific metabolic ratio. Simulations also predict that steady state dosing of modafinil may cause clinically relevant mDDIs resulting in reduced exposure to drugs metabolized by CYP3A4. This observation supports the current FDA classification of modafinil as a ‘moderate inducer’ of this enzyme (FDA, 2012).

## Conclusion

These data support consideration of the risk of clinically relevant mDDIs when co-administered modafinil with drugs that are primarily cleared by CYP 2C19 and 3A4 catalyzed metabolic pathways. In particular, given the major role of CYP3A4 in the clearance and metabolic activation of a myriad of drugs from various therapeutic classes including the oral contraceptive pill and orally administered non-cytotoxic anticancer drugs (e.g., erlotinib, pazopanib and sunitinib; Rowland et al., 2016) potential induction of CYP3A4 by modafinil should be considered when evaluating new indications for this drug such as the management of chemotherapy induced fatigue and cognitive impairment in order to avoid a potential decrease in therapeutic response for the co-prescribed drug that may result in therapeutic failure.

## Author Contributions

Participated in research design: AgR, AM, MS, and AdR. Conducted experiments: AgR and AdR. Performed data analysis: AgR, AM and AdR. Wrote or contributed to the writing of the manuscript: AgR, AM, AH, MS, and AdR.

## Conflict of Interest Statement

The authors declare that the research was conducted in the absence of any commercial or financial relationships that could be construed as a potential conflict of interest.
